# Intrawound injection of heroin as a drug delivery route among a cohort of people who inject drugs

**DOI:** 10.1002/hsr2.231

**Published:** 2021-01-18

**Authors:** Michael Anthony Huyck, Sarah Messmer, Stockton McQuade Mayer, Charles Yingling

**Affiliations:** ^1^ Department of Health Systems Science College of Nursing, University of Illinois at Chicago Chicago Illinois; ^2^ College of Medicine and Department of Infectious Disease University of Illinois at Chicago Chicago Illinois; ^3^ College of Medicine and Department of Pediatrics University of Illinois at Chicago Chicago Illinois; ^4^ College of Nursing and Department of Health Systems Science University of Illinois at Chicago Chicago Illinois

## 1 INTRODUCTION

Venous sclerosis and injection site vein loss are common complications experienced by people who inject drugs (PWID).^1^ When the preferred route of venous delivery has been exhausted, alternate practices of intramuscular and subcutaneous injection often result.^2^ Without access to preferred administration routes, PWID may engage in additional high‐risk practices such as injecting into chronic wound beds.^3^, ^4^

Acute and chronic wounds are commonplace among people who inject drugs. A variety of factors including the substance injected (cocaine, heroin, methamphetamines, pharmaceuticals) and utilizing poor injection‐related practices such as sharing, cleaning, and reusing syringes contribute to the risk of wound development.^5^, ^6^, ^7^, ^8^ Among clients frequenting syringe exchanges, current wound prevalence has been identified in as many as 10% to 20% of individuals.^6^, ^7^

The wound healing process encompasses angiogenesis and reepithelization which establishes a highly vascular network within the wound bed.^9^ Such highly vascular areas are effective routes of drug absorption including heroin. Multiple case studies have chronicled PWID directly injecting into chronic wounds as route of drug delivery.^3^, ^4^ Termed “shooter's patches,” individuals have cited quick and efficient drug absorption when injecting into wounds. Additionally, chronic wounds have been maintained by PWID for the sole purpose of injecting. Such practices are similarly resorted to when venous access is no longer a viable option.

## 2 METHODS

During October 2018 to August 2019, the University of Illinois at Chicago College of Nursing, College of Medicine and School of Public Health screened for injection practices among clients receiving wound care in once weekly half‐day medical clinic co‐located within a syringe exchange program. The medical clinic focused on providing primary care, medication‐assisted therapy, wound care, and infectious disease treatment for PWID. Clients presented on a walk‐in basis for harm reduction services and opted to receive medical care from onsite providers. All clients were screened for common injection practices such as intravenous, intramuscular, and subcutaneous injection. Clients presenting with acute or chronic wounds were specifically screened for intrawound injection practices as a treatment risk factor. Data for this descriptive study were collected retrospectively from a chart review of wound clinic clients. This work was reviewed by our institutional review board and determined to be exempt research.

## 3 RESULTS

**FIGURE 1:**
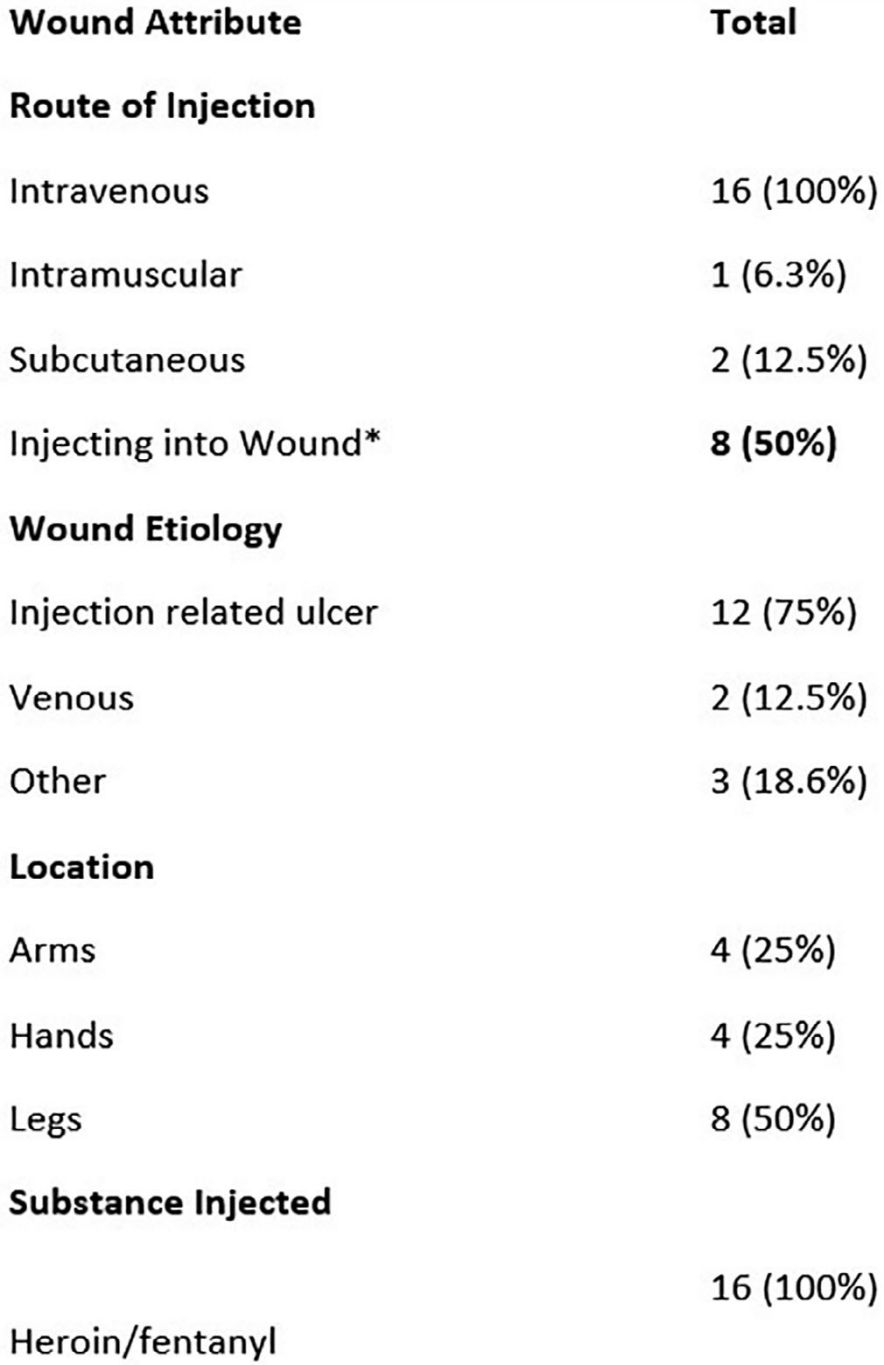
Demographics of wound clients October 2018 to August 2019

During the 10‐month pilot, the clinic cared for 85 unique clients. Wound‐specific clients represented 18.8% (*n* = 16) of all patients. Among wound clients, 50% (*n* = 8) reported intrawound injection practices (Figure 1). All wound clients (*n* = 16) reported intravenous injection practices, which were followed in frequency by directly injecting into the open wound bed (*n* = 8; 50%), subcutaneous (*n* = 2; 12.5%), and intramuscular injection (*n* = 1; 6.3%) (Figure 1). Some clients utilized multiple routes of drug delivery.

## 4 DISCUSSION

During development of our wound care program for PWID, our team considered intrawound injecting as a possible risk factor for treatment failure. While the practice has been previously mentioned in case studies, wider inquiry among PWID in wound‐related studies is generally missing. Therefore, we included it among our universal screening criteria to assess prevalence. Half of all wound clients endorsed intrawound injecting, which suggests more wide spread practice among PWID.

Among reasons mentioned for wound injecting were believing venous access was under the wound tissue and endorsement of an injection‐related “rush” often associated with intravenous injection. Clients with difficult intravenous access often utilized multiple routes of drug delivery including subcutaneous, intramuscular, and intrawound injection. Those utilizing intrawound injection reported a rapid onset and effect similar to intravenous injection.

In our practice, wounds that were used for injection purposes often presented with hypergranulation tissue, which also can also be a sign of infection or inflammation. Feelings of embarrassment often accompanied such admissions which further underscore a practice undertaken out of desperation. Potential limitations of this study include small sample size and geographic area, which may limit generalizability of the findings among PWID. Additionally, there is a lack of screening for intrawound injection practices in larger PWID studies which would corroborate the findings of this study.

### 4.1 Implications for practice

Given that half of wound clients endorsed intrawound injection, it represents a significant risk factor and requires screening when treating wounds within PWID. Wounds that exhibit treatment failure and deterioration after multiple visits should trigger a differential diagnosis of intrawound injection. The vascular tissue within the healing wound bed is an efficient drug delivery route, and clients with limited venous access may resort to maintaining chronic wounds for the purpose of injection.

Wound injection complicates the treatment process and highlights the need for intensive addiction treatment to prevent further complications. The perceived stigma and embarrassment expressed by patients in our practice underscore the importance of the therapeutic relationship as such practice was often revealed after multiple visits. Clients' disclosure of intrawound injection was often reluctant. Therefore, we recommend that all PWID be screened for this practice and that screening is done sensitively and tactfully. Even with proper identification and education about this practice, clients may continue to inject into their wounds. In such cases, treatment may require focus on harm reduction and palliation rather than cure.

## FUNDING

This work was funded by grants from the Hearst and Illinois Nurses Foundations. The funders did not have a role in the study design, implementation, or writing of this manuscript.

## CONFLICT OF INTEREST

The authors declare no conflicts of interest.

## AUTHOR CONTRIBUTIONS

Conceptualization: Michael Anthony Huyck

Formal analysis: Michael Anthony Huyck, Sarah Messmer

Funding acquisition: Michael Anthony Huyck, Stockton McQuade Mayer

Investigation: Michael Anthony Huyck, Sarah Messmer

Methodology: Michael Anthony Huyck, Charles Yingling

Project administration: Sarah Messmer, Stockton McQuade Mayer, Charles Yingling

Supervision: Sarah Messmer, Stockton McQuade Mayer, Charles Yingling

Writing ‐ original draft preparation: Michael Anthony Huyck

Writing ‐ review and editing: Charles Yingling

 All authors have read and approved the final version of the manuscript.

 Michael Anthony Huyck had full access to all of the data in this study and takes complete responsibility for the integrity of the data and the accuracy of the data analysis.

## TRANSPARENCY STATEMENT

Michael Anthony Huyck affirms that this manuscript is an honest, accurate, and transparent account of the study being reported; that no important aspects of the study have been omitted; and that any discrepancies from the study as planned (and, if relevant, registered) have been explained.

## Data Availability

The authors confirm that the data supporting the findings of this study are available within the article and its supplementary materials.
